# Cultural Reflections on Restrained Eating

**DOI:** 10.3389/fpsyg.2016.00205

**Published:** 2016-02-16

**Authors:** Adrian Meule

**Affiliations:** ^1^Department of Psychology, University of SalzburgSalzburg, Austria; ^2^Center for Cognitive Neuroscience, University of SalzburgSalzburg, Austria

**Keywords:** restrained eating, restraint scale, body mass index, dieting success, Asia, China

## Definition and measurement of restrained eating

Restrained eating refers to the intention to restrict food intake deliberately in order to prevent weight gain or to promote weight loss (Tuschl, 1990). There are several self-report instruments for the assessment of restrained eating, of which the three most commonly used are the *Restraint Scale* (RS; Herman and Mack, 1975; Herman and Polivy, 1975; Polivy et al., 1978), and the restraint subscales of the *Three-Factor Eating Questionnaire* (TFEQ, also *Eating Inventory*; Stunkard and Messick, 1985) and the *Dutch Eating Behavior Questionnaire* (DEBQ; Van Strien et al., 1986). Scores on these scales, however, do not necessarily reflect actual restriction of food intake or at least do this in a complex fashion (Stice et al., 2004, 2007, 2010; Van Strien et al., 2006). As most restrained eaters are not currently on a diet, they may be “best characterized as ‘weight watchers’ who are concerned about their food intake and try to limit intake, particularly of energy dense foods” (Lowe et al., 2013, p. 5).

## Restrained eating and body weight

While the restraint scales of the TFEQ and DEBQ have been found to identify rather successful restrained eaters (or, at least, high scores do not differentiate between successful and unsuccessful restrained eaters), the RS has been found to identify rather unsuccessful restrained eaters (Heatherton et al., 1988; Laessle et al., 1989; Van Strien, 1997, 1999). For example, individuals with high RS scores show a disinhibition, that is higher food intake, than those with low RS scores in various situations, for example after consumption of a preload, following food-cue exposure, or when self-regulatory resources are depleted (Stroebe, 2008; Stroebe et al., 2008). Furthermore, higher RS scores have consistently been found to be associated with higher body mass index (BMI; Ruderman, 1986; Stroebe, 2008; Stroebe et al., 2008). This relationship likely is bidirectional: restrained eating may predict weight gain (Van Strien et al., 2014), but having a high BMI may also lead to high restraint scores as the desire for losing weight and, thus, the intention to restrict food intake, increases with increasing body weight (Snoek et al., 2008).

Several studies have tried to differentiate successful and unsuccessful restrained eaters more clearly. For example, Westenhoefer et al. (1994) could show that eating restraint as measured with the TFEQ was only associated with higher laboratory food intake in combination with high scores on the disinhibition subscale of the TFEQ. In other studies, the *concern for dieting* subscale of the RS was used in combination with a self-report measure of perceived dieting success (Meule et al., 2012b; Stroebe et al., 2013). Expectedly, unsuccessful restrained eaters had an higher BMI than successful restrained eaters (Meule et al., 2012a). To conclude, there is ample evidence, which shows that unsuccessful restrained eating—as assessed with either the RS or with a combination of restraint and other scales—is related to higher BMI.

## Restrained eating and body weight in Asia

In a recent article, Kong et al. (2015) investigated individuals from Chongqing in Southwest China and used a combination of different questionnaire scores to classify participants as successful and unsuccessful restrained eaters. Specifically, participants with high scores on the restraint subscale of the DEBQ, but low scores on the emotional and external eating subscales of the DEBQ and low scores on the disinhibition subscale of the TFEQ were classified as successful restrained eaters, while participants with high scores on all these measures were classified as unsuccessful restrained eaters. Of note, groups did not differ in BMI and, descriptively, the successful restrained eaters even had a higher mean BMI (although far from statistical significance).

In two other recent articles with participants from the same city, the RS was used to classify participants as restrained (score > 15) and unrestrained eaters (score < 12) (Dong et al., 2014; Chen et al., 2016). Here, groups did not differ in BMI as well. Both findings stand in contrast to decades of research in Western societies, which showed that RS scores are positively associated with BMI and with studies that more explicitly differentiated between successful and unsuccessful restrained eaters (see above). In the USA, the Netherlands, Germany, and other countries, the relationship between RS scores and BMI is at least medium-sized (Papies et al., 2008; Stroebe, 2008; Meule et al., 2011, 2012c). In yet another study by the same group, however, restrained eating was indeed positively correlated with BMI, but this was based on the restraint subscale of the DEBQ (Dong et al., 2015).

There may be several possible explanations why the association between restrained eating and body mass in Asia is, at least, inconsistent. It should be noted, however, that these are mere speculations without sufficient evidence to support or refute them. A first one may be general differences in nutrition between individuals from Asia and Western countries, with the former having a healthier, less energy-dense diet (Lv and Cason, 2004; Guendelman et al., 2011). It may be speculated that “unsuccessful” restrained eaters in Asia do not gain weight because even in the absence of successful restraint they still do not consume a large amount of energy. Another explanation may be changes in item wording as a result of translating the scale to an unrelated language or that some statements in restraint questionnaires have a different meaning in Asia. Also, as the RS includes questions on weight fluctuations, it may be particularly susceptible to cultural differences related to body weight and –composition. Specifically, the response categories, which specify certain values (in pounds) may be inappropriate for Asian populations. It is known that Asian populations have a lower BMI and different associations between BMI and percentage of body fat than Caucasians (WHO Expert Consultation, 2004). A higher percent body fat at low BMI in Asian populations can be partly explained by differences in trunk-to-leg-length ratio and slenderness (Deurenberg et al., 2002). It seems that there also differences in the BMI-body fat relationship between different Asian populations and even between different Chinese groups (Deurenberg et al., 2002). These differences may also influence the relationship between restrained eating and BMI. For example, in a study with Hong Kong Chinese adolescents, there was indeed a positive association between RS scores and BMI (Mak and Lai, 2012; Lai et al., 2013). Nonetheless, even when such anthropometric differences between people with different ethnic backgrounds exist, one would expect that the relationship between restrained eating and BMI would be the same *within* each group. Thus, if and how ethnic differences in body composition and body build can account for differences in the relationship between restrained eating and BMI remains to be elucidated.

## Conclusion

Kong et al. (2015) aimed to investigate the neural and behavioral correlates of successful and unsuccessful restrained eating. However, if groups have the same body mass, what is it that makes unsuccessful restrained eaters unsuccessful? Surely, a person's success or failure in eating restraint cannot be judged by using this person's BMI alone. For example, an overweight person might be indeed a rather successful restrained eater if this person's BMI would be even higher in the absence of an effort to lose weight (cf. Lowe, 2015). The complete absence of a relationship between unsuccessful restraint and BMI, however, is unusual. Thus, this commentary argues that restrained eating, for example as measured with the RS, may not represent the same phenomenon in (at least some) Asian populations as it does in Western societies. Thus, caution should be exercised when interpreting studies on the mechanisms of successful and unsuccessful restrained eating as the cultural background appears to be a crucial aspect to consider.

### Conflict of interest statement

The author declares that the research was conducted in the absence of any commercial or financial relationships that could be construed as a potential conflict of interest.

## References

[B1] Chen S. Dong D. Jackson T. Su Y. Chen H. (2016). Altered frontal inter-hemispheric resting state functional connectivity is associated with bulimic symptoms among restrained eaters. Neuropsychologia 81, 22–30. 10.1016/j.neuropsychologia.2015.06.036 26160289

[B2] Deurenberg P. Deurenberg-Yap M. Guricci S. (2002). Asians are different from Caucasians and from each other in their body mass index/body fat per cent relationship. Obes. Rev. 3, 141–146. 10.1046/j.1467-789X.2002.00065.x 12164465

[B3] Dong D. Jackson T. Wang Y. Chen H. (2015). Spontaneous regional brain activity links restrained eating to later weight gain among young women. Biol. Psychol. 109, 176–183. 10.1016/j.biopsycho.2015.05.003 26004091

[B4] Dong D. Lei X. Jackson T. Wang Y. Su Y. Chen H. (2014). Altered regional homogeneity and efficient response inhibition in restrained eaters. Neuroscience 266, 116–126. 10.1016/j.neuroscience.2014.01.062 24513387

[B5] Guendelman M. D. Cheryan S. Monin B. (2011). Fitting in but getting fat: identity threat and dietary choices among U.S. immigrant groups. Psychol. Sci. 22, 959–967. 10.1177/0956797611411585 21653909

[B6] Heatherton T. F. Herman C. P. Polivy J. King G. A. McGree S. T. (1988). The (mis)measurement of restraint: an analysis of conceptual and psychometric issues. J. Abnorm. Psychol. 97, 19–28. 10.1037/0021-843X.97.1.19 3280636

[B7] Herman C. P. Mack D. (1975). Restrained and unrestrained eating. J. Pers. 43, 647–660. 10.1111/j.1467-6494.1975.tb00727.x 1206453

[B8] Herman C. P. Polivy J. (1975). Anxiety, restraint, and eating behavior. J. Abnorm. Psychol. 84, 666–672. 10.1037/0021-843X.84.6.666 1194527

[B9] Kong F. Zhang Y. Chen H. (2015). Inhibition ability of food cues between successful and unsuccessful restrained eaters: a two-choice oddball task. PLoS ONE 10:e0120522. 10.1371/journal.pone.0120522 25886063 PMC4401785

[B10] Laessle R. G. Tuschl R. J. Kotthaus B. C. Pirke K. M. (1989). A comparison of the validity of three scales for the assessment of dietary restraint. J. Abnorm. Psychol. 98, 504–507. 10.1037/0021-843X.98.4.504 2592686

[B11] Lai C.-M. Mak K.-K. Pang J. S. Fong S. S. M. Ho R. C. M. Guldan G. S. (2013). The associations of sociocultural attitudes towards appearance with body dissatisfaction and eating behaviors in Hong Kong adolescents. Eat. Behav. 14, 320–324. 10.1016/j.eatbeh.2013.05.004 23910774

[B12] Lowe M. R. (2015). Dieting: proxy or cause of future weight gain? Obes. Rev. 16, 19–24. 10.1111/obr.12252 25614200

[B13] Lowe M. R. Doshi S. D. Katterman S. N. Feig E. H. (2013). Dieting and restrained eating as prospective predictors of weight gain. Front. Psychol. 4:577. 10.3389/fpsyg.2013.00577 24032024 PMC3759019

[B14] Lv N. Cason K. L. (2004). Dietary pattern change and acculturation of Chinese Americans in Pennsylvania. J. Am. Diet. Assoc. 104, 771–778. 10.1016/j.jada.2004.02.032 15127063

[B15] Mak K.-K. Lai C.-M. (2012). Assessment of dietary restraint: psychometric properties of the revised restraint scale in Hong Kong Adolescents. Int. J. Behav. Med. 19, 199–207. 10.1007/s12529-011-9161-x 21553306 PMC3358557

[B16] Meule A. Lukito S. Vögele C. Kübler A. (2011). Enhanced behavioral inhibition in restrained eaters. Eat. Behav. 12, 152–155. 10.1016/j.eatbeh.2011.01.006 21385646

[B17] Meule A. Lutz A. Vögele C. Kübler A. (2012a). Food cravings discriminate differentially between successful and unsuccessful dieters and non-dieters. Validation of the Food Craving Questionnaires in German. Appetite 58, 88–97. 10.1016/j.appet.2011.09.010 21983051

[B18] Meule A. Papies E. K. Kübler A. (2012b). Differentiating between successful and unsuccessful dieters. Validity and reliability of the Perceived Self-Regulatory Success in Dieting Scale. Appetite 58, 822–826. 10.1016/j.appet.2012.01.028 22329988

[B19] Meule A. Vögele C. Kübler A. (2012c). Restrained eating is related to accelerated reaction to high caloric foods and cardiac autonomic dysregulation. Appetite 58, 638–644. 10.1016/j.appet.2011.11.023 22142510

[B20] Papies E. K. Stroebe W. Aarts H. (2008). Healthy cognition: processes of self-regulatory success in restrained eating. Person. Soc. Psychol. Bull. 34, 1290–1300. 10.1177/0146167208320063 18596220

[B21] Polivy J. Herman C. P. Warsh S. (1978). Internal and external components of emotionality in restrained and unrestrained eaters. J. Abnorm. Psychol. 87, 497–504. 10.1037/0021-843X.87.5.497 701602

[B22] Ruderman A. J. (1986). Dietary restraint: a theoretical and empirical review. Psychol. Bull. 99, 247–262. 10.1037/0033-2909.99.2.247 3515384

[B23] Snoek H. M. Van Strien T. Janssens J. Engels R. (2008). Restrained eating and BMI: a longitudinal study among adolescents. Health Psychol. 27, 753–759. 10.1037/0278-6133.27.6.753 19025271

[B24] Stice E. Cooper J. A. Schoeller D. A. Tappe K. Lowe M. R. (2007). Are dietary restraint scales valid measures of moderate- to long-term dietary restriction? Objective biological and behavioral data suggest not. Psychol. Assess. 19, 449–458. 10.1037/1040-3590.19.4.449 18085937

[B25] Stice E. Fisher M. Lowe M. R. (2004). Are dietary restraint scales valid measures of acute dietary restriction? Unobtrusive observational data suggest not. Psychol. Assess. 16, 51–59. 10.1037/1040-3590.16.1.51 15023092

[B26] Stice E. Sysko R. Roberto C. A. Allison S. (2010). Are dietary restraint scales valid measures of dietary restriction? Additional objective behavioral and biological data suggest not. Appetite 54, 331–339. 10.1016/j.appet.2009.12.009 20006662 PMC2830309

[B27] Stroebe W. (2008). Restrained eating and the breakdown of self-regulation, in Dieting, Overweight, and Obesity - Self-Regulation in a Food-Rich Environment, ed Stroebe W. (Washington, DC: American Psychological Association), 115–139.

[B28] Stroebe W. Mensink W. Aarts H. Schut H. Kruglanski A. W. (2008). Why dieters fail: testing the goal conflict model of eating. J. Exp. Soc. Psychol. 44, 26–36. 10.1016/j.jesp.2007.01.005

[B29] Stroebe W. Van Koningsbruggen G. M. Papies E. K. Aarts H. (2013). Why most dieters fail but some succeed: a goal conflict model of eating behavior. Psychol. Rev. 120, 110–138. 10.1037/a0030849 23230892

[B30] Stunkard A. J. Messick S. (1985). The Three-Factor Eating Questionnaire to measure dietary restraint, disinhibition and hunger. J. Psychosom. Res. 29, 71–83. 10.1016/0022-3999(85)90010-8 3981480

[B31] Tuschl R. J. (1990). From dietary restraint to binge eating: some theoretical considerations. Appetite 14, 105–109. 10.1016/0195-6663(90)90004-R 2186700

[B32] Van Strien T. (1997). Are most dieters unsuccessful? An alternative interpretation of confounding of success and failure in the measurement of restraint. Euro. J. Psychol. Assess. 13, 186–194. 10.1027/1015-5759.13.3.186

[B33] Van Strien T. (1999). Success and failure in the measurement of restraint. Int. J. Eat. Disord. 25, 441–449. 10202655 10.1002/(sici)1098-108x(199905)25:4<441::aid-eat9>3.0.co;2-b

[B34] Van Strien T. Engels R. Van Staveren W. Herman C. P. (2006). The validity of dietary restraint scales: comment on Stice et al. (2004). Psychol. Assess. 18, 89–94. 10.1037/1040-3590.18.1.89 16594816

[B35] Van Strien T. Frijters J. E. R. Bergers G. P. A. Defares P. B. (1986). The Dutch Eating Behavior Questionnaire (DEBQ) for assessment of restrained, emotional, and external eating behavior. Int. J. Eat. Disord. 5, 295–315.

[B36] Van Strien T. Herman C. P. Verheijden M. W. (2014). Dietary restraint and body mass change. A 3-year follow up study in a representative Dutch sample. Appetite 76, 44–49. 10.1016/j.appet.2014.01.015 24480668

[B37] Westenhoefer J. Broeckmann P. Münch A.-K. Pudel V. (1994). Cognitive control of eating behaviour and the disinhibition effect. Appetite 23, 27–41. 10.1006/appe.1994.1032 7826055

[B38] WHO Expert Consultation (2004). Appropriate body-mass index for Asian populations and its implications for policy and intervention strategies. Lancet 363, 157–163. 10.1016/S0140-6736(03)15268-3 14726171

